# The Impact of Empowering Leadership on the Job Crafting of Knowledge Employees: A Moderated Mediating Effect Model

**DOI:** 10.3390/bs16010117

**Published:** 2026-01-14

**Authors:** Yu Mao, Quan Fang, Chunyan Jiang, Huabin Wu

**Affiliations:** 1 School of Business, Nanjing University, Nanjing 210008, China; yumao@smail.nju.edu.cn (Y.M.); 602022020077@nju.edu.cn (H.W.); 2 School of Business, Nanjing University of Science and Technology ZiJin College, Nanjing 210094, China

**Keywords:** empowering leadership, job crafting, role breadth self-efficacy, learning goal orientation, social cognitive theory

## Abstract

Empowering leadership can provide more resource support for organizations and better match the characteristics of current knowledge employees, such as a high demand for autonomy and pursuit of value diversification. However, existing literature has not fully clarified the specific cognitive transmission mechanisms linking empowering leadership to knowledge workers’ job crafting, nor has it sufficiently examined the boundary conditions of this relationship under specific individual traits. This study aimed to explore the impact of empowering leadership on knowledge employees’ job crafting by constructing a moderated mediation model. This study introduces role breadth self-efficacy as a mediating variable and learning goal orientation as a moderating variable and collects questionnaire data to investigate the underlying mechanisms among 338 knowledge employees. Empowering leadership has a positive effect on knowledge employees’ job crafting. Role breadth self-efficacy mediates the relationship between empowering leadership and job crafting. Learning goal orientation positively moderated the impact of empowering leadership on role breadth self-efficacy. Building an empowering leadership model with empowerment and psychological safety at its core can stimulate employees’ job crafting. Role breadth self-efficacy can be improved by challenging tasks and systematic training. The implementation of differentiated management based on learning-goal orientation strengthens the empowerment effect. These measures provide feasible paths for organizations to drive adaptive changes.

## 1. Introduction

The wave of global digital transformation and China’s high-quality economic development strategy have led to the comprehensive arrival of the era of the knowledge economy. The siphoning effect of emerging industries, represented by artificial intelligence and big data, on highly skilled talent has continued to increase. As the core carrier of technological innovation, the professional behavior patterns of knowledge workers have undergone structural changes. Their strong autonomy demands and diversified value paths have led to new requirements for organizational management models. A generational shift in the workforce leads to a transition in value. When selecting employers, Gen Z employees increasingly prioritize professional value alignment and career development potential. Compensation levels are no longer the sole measure of career value; more individuals seek the dual enhancement of personal fulfillment and societal contribution through their professional platforms.

Unlike traditional operational employees, whose tasks are often standardized and routine, knowledge workers are defined by their high cognitive complexity and a strong need for autonomy (Drucker, 1999). Their performance relies heavily on intrinsic motivation, rather than adherence to strict directives. Consequently, the autonomy and participative decision-making provided by empowering leadership are not merely supportive factors but are essential structural antecedents that align with the professional nature of knowledge workers.

Therefore, proactively adjusting the content and meaning of work is crucial for cultivating top-down initiatives among knowledge workers and enabling them to realize their self-worth (Wrzesniewski & Dutton, 2001). This type of job crafting promotes innovative and proactive behaviors (Tho, 2022), enhances employees’ sense of meaning at work (van Wingerden et al., 2017) and job engagement (Bakker et al., 2012), and significantly increases career success. Current research predominantly examines job crafting as an antecedent variable, focusing on individual and situational factors such as personality traits (Bateman & Crant, 1993) and job stress (Füllemann et al., 2015). However, studies on the impact of leadership style, particularly empowering leadership, on job crafting remain scarce. Empowering leadership provides employees with autonomy and space aligned with the work values of knowledge workers. Cognitively, empowering leadership enhances subordinates’ role breadth self-efficacy (Den Hartog & Belschak, 2012; Parker, 1998). Research on the interaction effect between personal traits and leadership style is relatively scarce. Individual differences play a key moderating role in leadership effects. As a key intrinsic motivation for pursuing competence, a learning-goal orientation may amplify the promotional effect of empowering leadership on job crafting.

This study focuses on knowledge workers and employs the social cognitive theory as its theoretical foundation to construct a three-dimensional mechanism model: leadership empowerment, cognitive activation, and behavioral restructuring. By taking role breadth self-efficacy as the mediating variable and learning goal orientation as the moderating variable, the influence mechanism of empowering leadership on job crafting behavior and related boundary conditions were explored. The possible contributions of this study are as follows.

First, it refines the theoretical framework of the leadership styles that influence job crafting. Research on the antecedents of job crafting has focused primarily on dimensions, such as task characteristics, individual traits, and organizational environments (Parker, 1998), whereas the mechanism of leadership style as a key contextual variable remains underexplored. This study constructs a moderated mediation model and presents a novel theoretical perspective.

Second, this study deepens the research on social cognitive theory. By revealing the formation mechanism of knowledge workers’ job-crafting behaviors, this study focuses on the transmission effect of individual efficacy beliefs between environmental factors and employees’ active behaviors, verifies the applicability of social cognitive theories in explaining employees’ role expansion behaviors in organizational contexts, and provides new evidence for the contextualized development of theories.

## 2. Literature Review

### 2.1. Organizational Behavior of Empowering Leadership

Empowering leadership is an important concept in the field of organizational behavior and leadership research, and its connotations have gradually deepened with the development of theories. Empowering leadership significantly enhances employees’ sense of happiness, self-efficacy, and work mood from multiple dimensions by creating a supportive environment and empowerment (Hao et al., 2018; Kim & Beehr, 2018; Schaufeli & Bakker, 2004) and can also reduce job burnout by granting employee autonomy through empowerment. Empowering leadership can also positively affect team performance. When managers provide teams with space for autonomous decision-making, they prompt members to form complementary cognitive networks, and each member can not only clearly recognize the professional strengths of their colleagues but also establish cross-functional collaboration trust, achieving efficient integration of knowledge and thereby enhancing team performance. Leadership behavior jointly drives team effectiveness through emotional and cognitive synergies (DeChurch & Mesmer-Magnus, 2010).

From a dynamic perspective, empowering leadership enhances employees’ overall adaptability in complex situations. This increases employees’ role breadth self-efficacy, which is further strengthened by the trust-based environment and the systematic functional guidance provided (Dirks & de Jong, 2022). When managers adopt a management style that combines humanistic care with engaging elements, it effectively promotes employees’ self-efficacy development in the role expansion dimension (Rodrigues & Rebelo, 2023). Helping role breadth self-efficacy plays a key mediating role between leadership and employee innovation behaviors (Rodrigues & Rebelo, 2023).

### 2.2. Influence of Leadership Styles on Job Crafting

Job crafting refers to employees’ ability to achieve a dynamic alignment between themselves and their roles by proactively adjusting their job content and form. Self-regulation occurs through three dimensions: expanding task boundaries, restructuring relational networks, and reshaping cognitive meaning (Wrzesniewski & Dutton, 2001). Subsequent studies have defined job fit as a dynamic process in which employees enhance their job fit by supplementing their job resources, increasing challenging demands, and reducing hindering demands. At the individual level, positive factors such as facilitative regulatory focus and achievement motivation stimulate self-motivation and goal orientation, prompting employees to proactively optimize job resources and demands, thereby positively influencing facilitative job crafting (van Wingerden et al., 2017; Zhang et al., 2025). At the situational level, research reveals that by integrating job characteristic elements (Meyer et al., 2014), leaders can significantly enhance knowledge workers’ job crafting through increased perceptions of psychological empowerment and professional belonging (Bharadwaja & Tripathi, 2020; Cenciotti et al., 2017; Guo et al., 2023; Messmann, 2023; Nguyen et al., 2024; Seo, 2023). Employees’ sense of group belonging, formed through organizational socialization, can effectively translate into motivation (Saks & Gruman, 2014) and transform challenging work stress into self-efficacy (Füllemann et al., 2015).

A preliminary consensus has emerged regarding the relationship between leadership and job crafting. However, the dynamic interaction mechanism between the two remains underexplored. Job crafting requires autonomy and resource support, as well as the conditions that empowering leaders can fulfill through institutional arrangements and emotional engagement. Empowering leadership influences employee behavior through multiple dimensions, such as fostering innovative behavior and organizational citizenship behavior (Arnold et al., 2000; Auh et al., 2014), but its cognitive transmission pathways remain poorly understood. Core variables in knowledge worker research, such as learning goal orientation, role breadth self-efficacy (Den Hartog & Belschak, 2012), and organizational context (Fischer & Sitkin, 2023), are yet to be systematically integrated into a theoretical framework with leadership styles. Moreover, there is no comprehensive explanation of how empowering leadership supports employees’ behavioral restructuring. Therefore, on the basis of social cognitive theory, this study constructs a mediating path of “empowering leadership—role breadth self-efficacy—job crafting” and introduces the moderating effect of learning goal orientation. This study integrates the dual driving paths of the structural and psychological empowerment inherent in empowering leadership. Furthermore, it reveals the key cognitive transmission mechanism via role breadth self-efficacy and clarifies how learning-goal orientation, as a boundary condition, dynamically shapes these leadership effects. This model compensates for the insufficient integration of the mediating and moderating mechanisms in the literature and provides a more powerful explanatory tool for the active behavior of knowledge workers in complex organizational environments.

### 2.3. Research Hypotheses

#### 2.3.1. Direct Effects

Empowering leadership stimulates employee initiative through decentralization, empowerment, and resource support (Bakker et al., 2007). Empowering leadership provides employees with opportunities to actively change their work content, processes, or cognition by creating a supportive environment in work and organizational decision making (Conger & Kanungo, 1988). Research indicates that enhancement of organizational resource support encourages employees to proactively adjust their job content (Bakker et al., 2012). Empowering a leadership model not only needs to establish a trust relationship through emotional interaction but also needs to provide necessary resource assistance to ensure work autonomy (Arnold et al., 2000). Empowering leadership has a significant positive effect on employees’ self-efficacy, providing them with more psychological support (Kim & Beehr, 2018) and creating a highly supportive organizational and psychologically safe environment. This precisely conforms to the process of job crafting, in which employees adjust their work resources and requirements to enhance their job fit.

According to the social cognitive theory, empowering leadership can guide job crafting in two ways. On the one hand, social cognitive theory holds that the environment has a shaping effect on individual behavior. Reasonable empowerment behaviors objectively increase structural work resources and expand employees’ behavioral boundaries. Resources, such as tools and information provided by leaders, lower the threshold for job crafting and enable employees to experiment with opportunities afforded by empowerment behaviors. However, individuals can act as active agents of change in the process of self-development, self-adaptation, and self-renewal. Leaders’ encouraging attitudes influence employees’ expectations of behavioral outcomes, reduce the risk of failure in behavioral consequences, and motivate their attempts. In summary, empowering leadership creates a favorable working and psychological environment for employees and promotes job-crafting behaviors. This study proposes the following hypothesis:

**Hypothesis** **1.** *Empowering leadership has a significantly positive effect on job crafting.*

Role breadth self-efficacy, as an extension of self-efficacy in the field of organizational behavior, refers specifically to individuals’ self-confidence in completing cross-functional comprehensive tasks (Parker, 1998). Emphasis is placed on the self-assessment of employees’ role extension behavior. Empirical studies have shown that employees with high role-breadth self-efficacy perceive their ability to go beyond completing the required comprehensive tasks. Empowering leadership can enhance individuals’ sense of self-efficacy. Management empowerment behavior enables employees to develop positive perceptions of their capabilities by establishing a decision-making participation mechanism (Ahearne et al., 2005) and system of equal rights and responsibilities (Conger & Kanungo, 1988). When managers simultaneously implement information sharing, responsibility matching, and development guidance (Arnold et al., 2000; Kim & Beehr, 2018), employees’ assessments of their potential for cross-boundary performance show a positive response to stepwise improvement. Studies have confirmed that leadership style can significantly affect employees’ role breadth self-efficacy (Rodrigues & Rebelo, 2023). Leaders’ trust and support create a trusting and supportive environment for employees and enhance their role-breadth self-efficacy. On the other hand, empowering leadership enables employees to have a sense of control over their work by endowing them with work autonomy and participation in decision-making, which is also an important means to improve their role breadth self-efficacy (Parker, 1998). Based on the above analysis, this study proposes the following hypothesis:

**Hypothesis** **2.** *Empowering leadership has a significantly positive effect on role breadth self-efficacy.*

Social cognitive theory points out that psychological cognition can drive the generation of behavioral choices. Role breadth self-efficacy, as an individual’s cognitive evaluation system for cross-border duty performance ability, affects work behavior through the three-dimensional path of “expectation-motivation-regulation.” First, when the organizational environment transmits empowerment signals through empowerment management or resource support, individuals gradually form cognitive expectations of transboundary task competency, based on past experience and situational evaluation. Individuals with high self-efficacy systematically assess their competence potential in unconventional tasks. This cognitive advantage prompts individuals to actively seek breakthrough tasks rather than avoid risks (Mumford et al., 2003). This trait can transform role ambiguity into the identification of innovation opportunities through the mechanism of stress-cognitive reconstruction, thereby catalyzing the continuous output of active adaptive behaviors (Lord & Emrich, 2000). Thus, role breadth self-efficacy promotes the growth and development of individuals and enables employees to engage in more proactive and extra-role behaviors. As a proactive employee behavior, job crafting provides grounds to infer that role breadth self-efficacy positively influences job crafting. Therefore, this study proposes the following hypotheses:

**Hypothesis** **3.** *Role breadth self-efficacy positively affects employees’ job crafting.*

According to Social Cognitive Theory (Bandura, 2001), environmental factors primarily influence human behavior through cognitive self-regulation mechanisms. Empowering leadership acts as a critical contextual resource by providing decision-making autonomy and support (Kim & Beehr, 2021). However, to drive proactive behavior, external structural support must be internalized into PsyCap. Role breadth self-efficacy serves as an essential cognitive bridge; it transforms the objective resources provided by leaders into a subjective belief in one’s capability to handle broader responsibilities. Consequently, enhanced self-efficacy motivates employees to transcend rigid role descriptions and engage in job crafting. Thus, this study proposes the following hypothesis:

**Hypothesis** **4.** *Role breadth self-efficacy mediates the relationship between empowering leadership and job crafting.*

#### 2.3.2. Moderating Effect

Learning goal orientation, as an individual trait, reflects an individual’s expectations for “mastering new skills, deeply understanding new things, adapting to new environments, and enhancing one’s own competence” (VandeWalle, 1997). Individuals with a learning goal-oriented mindset are more willing to accept challenging goals, and have strong intrinsic motivation and autonomy in their work (Van Yperen & Hagedoorn, 2003). From the perspective of individual ability cognition, learning goal-oriented individuals adhere to the dynamic development concept of ability and tend to proactively seek challenging tasks to expand their ability boundaries and cope better with work difficulties (Dweck, 1986). The research object of this study is knowledge workers, whose core characteristics are continuous knowledge updating and innovative applications, which are more relevant to learning-goal orientation.

Empowering leadership significantly enhances employees’ role breadth self-efficacy by granting them autonomy in decision-making, resource support, and psychological safety. However, this positive effect does not equally apply to all employees. Learning goal orientation, a core trait that measures individuals’ intrinsic motivation to learn, determines how employees interpret and utilize leadership empowerment behaviors. For employees with strong learning-goal orientation, the autonomous space provided by empowering leadership is regarded as an opportunity for ability development, and they are more likely to actively explore new tasks and integrate cross-domain knowledge. Conversely, employees with low learning goal orientation may view empowerment as a performance pressure or an additional burden, lacking the motivation to actively expand their role boundaries. This diminishes the enhancing effect of empowering leadership. Consequently, this study proposes the following hypothesis:

**Hypothesis** **5.** *Learning-goal orientation positively moderates the effect of empowering leadership on knowledge workers’ role breadth self-efficacy.*

Based on the triadic interaction model grounded in social cognitive theory, this study proposed that learning-goal orientation exerts a significant moderating effect on the mediating pathway. Specifically, learning goal orientation, as an individual trait, affects the positive effect of empowering leadership on role breadth self-efficacy and regulates the transmission strength of the whole mediation mechanism. For employees with a strong learning-goal orientation, intrinsic motivation centers on enhancing their capabilities. They perceived leadership empowerment as a developmental opportunity, proactively leveraging an autonomous decision-making space and resource support to attempt cross-boundary tasks. This positive cognition amplifies the enhancement of role breadth self-efficacy, leading employees to evaluate their comprehensive capabilities more optimistically and thereby engage more actively in job-crafting behaviors. In contrast, employees with low learning goal orientation tend to interpret empowerment as a burden or risk due to their fixed ability, which leads to limited promotion of role breadth self-efficacy, thereby attenuating the indirect effect of empowering leadership on job crafting. Thus, this study proposes the following hypothesis:

**Hypothesis** **6.** *Learning goal orientation significantly moderate the mediating path of role-breadth self-efficacy between empowering leadership and job crafting.*

**Figure 1:**
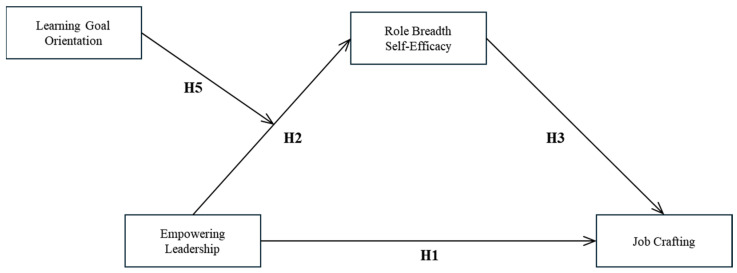
The conceptual model. Note: H1, H2, H3, and H5 represent direct and moderating paths as indicated. H4 (not shown on paths) predicts the mediating role of role breadth self-efficacy. H6 predicts the moderated mediation effect of learning goal orientation.

In summary, this study used social cognitive theory to construct a theoretical model of the four variables of empowering leadership—role breadth self-efficacy, learning goal orientation, and job crafting—to explore the influence mechanism of empowering leadership on job crafting, as shown in Figure 1.

## 3. Methodology

### 3.1. Data Collection Procedure

**Figure 2:**
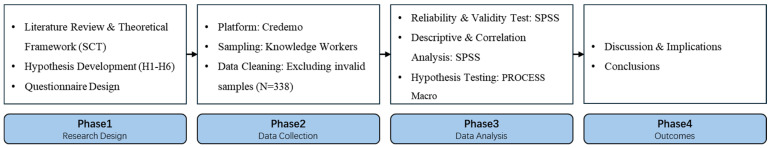
Flowchart of the research process.

This study employed a cross-sectional design to investigate the mechanism through which empowering leadership influences job-crafting behavior, as illustrated in the research process flowchart (Figure 2). Data were collected using the digital questionnaire platform CreDemo. To ensure data quality, we employed a systematic sampling approach targeting workplace professionals. In total, 396 responses were obtained. After rigorous data-cleaning procedures, excluding responses with invalid time logs (e.g., answering too quickly), and incomplete data, 338 valid observations were confirmed, resulting in an effective response rate of 85.4%.

### 3.2. Participants

The final sample comprised 338 employees. The demographic characteristics revealed a relatively balanced sex ratio (52.1% male and 47.9% female). The sample was knowledge-intensive in terms of educational background: 42% held a bachelor’s degree and 16.3% held a master’s degree or above. Regarding hierarchical positions, the distribution included grassroots employees (58.6%), middle-level managers (17.8%), and senior managers (23.7%).

### 3.3. Instruments

Empowering leadership was measured based on H. Wang et al.’s (2008) six-dimensional empowering leadership scale (H. Wang et al., 2008), which comprises six items covering decision-making empowerment, information sharing, competency recognition, innovation support, resource provision, and reverse-scored control behaviors. Sample items include “My leader empowers me with the decision-making authority needed to complete my work” and “My leader habitually micromanages work details throughout the process (reverse-scored item).” The reliability analysis yielded a Cronbach’s alpha of 0.897, indicating good reliability and suitability for measuring leadership delegation behaviors.

The measurement of job crafting employed Z. Wang et al.’s (2019) five-dimensional job crafting scale (Z. Wang et al., 2019), comprising six items that assess employees’ proactive behavior in adjusting job content, processes, skill integration, enhancing meaning, and expanding responsibilities. Sample items included “I proactively adjust my job content to better align with my personal interests” and “I typically take on challenging tasks beyond my job scope.” Cronbach’s alpha was 0.913, which effectively reflects individuals’ proactive job redesign behaviors.

The measurement of role breadth self-efficacy employed Parker et al. (2006) revised role breadth self-efficacy scale (Parker et al., 2006), comprising five items that assessed employees’ confidence in resolving issues beyond their responsibilities, their ability to suggest process improvements, and their effectiveness in cross-departmental collaboration. Sample items included, “I can propose effective suggestions for process optimization” and “I feel inadequate in resolving issues outside my scope of responsibility (reverse-scored).” The reliability of Cronbach’s alpha was 0.814, indicating stable reliability.

The measurement of learning goal orientation refers to VandeWalle’s (1997) Learning Goal Orientation Scale (VandeWalle, 1997), which consists of five items and measures an individual’s acceptance of learning-oriented tasks, awareness of challenge transformation, and willingness to improve skills. Sample items include “I am willing to accept new tasks that can enhance my ability” and “I pay more attention to the completion of work rather than the improvement of personal ability (reverse scoring).” Cronbach’s alpha for reliability was 0.87, which satisfied the research requirements.

### 3.4. Data Analysis Procedure

Data analysis was performed using SPSS 24.0. Prior to hypothesis testing, control variables were coded to ensure statistical validity, gender was dummy coded (0 = male, 1 = female), age and working years were measured as continuous quantitative variables, and educational background and position level, being ordinal in nature, were treated as continuous covariates to preserve rank-order information. First, we performed Harman’s single-factor test to check for a common method bias. Second, we calculated descriptive statistics and Pearson’s correlation coefficients to examine the relationships among the variables. Third, to rigorously test the mediation (H4) and moderated mediation (H6) hypotheses, we employed the PROCESS macro (Hayes, 2017) in SPSS. Model 4 was utilized for the mediation analysis, and Model 7 was used for the moderated mediation analysis. The bias-corrected bootstrap method with 5000 resamples was applied to estimate confidence intervals (CIs) for significance testing.

## 4. Results

### 4.1. Common Variance Test

Given that this study employed a questionnaire method for data collection, Harman’s single-factor analysis technique was used to examine the potential common method bias. In the unrotated exploratory factor analysis, the first principal component accounted for 39.242% of the variance, falling below the conventional 40% discriminant threshold (Podsakoff et al., 2003). This finding indicates that the covariant relationship of variables is driven mainly by the characteristics of the construct itself rather than by the systematic bias of the measurement methods; therefore, there is no serious common method bias in the data of this study.

### 4.2. Descriptive Statistics and Correlation Analysis

Table 1 presents the descriptive statistics and bivariate association matrices of the core variables. Quantitative analysis revealed the following key paths: there was a positive correlation between empowering leadership and job crafting behaviors (r = 0.51, *p* < 0.01); empowering leadership was significantly positively correlated with role breadth self-efficacy (r = 0.43, *p* < 0.01); and there was a significant correlation between role breadth self-efficacy and job crafting behavior (r = 0.34, *p* < 0.01).

### 4.3. Hypothesis Testing

To rigorously test the mediation and moderated mediation effects, we adopted PROCESS macro Model 4 for mediation and Model 7 for moderated mediation (Hayes, 2017) rather than the traditional causal steps approach (Baron & Kenny, 1986). The core advantage of this framework lies in its ability to directly estimate the indirect effect through bias-corrected bootstrapping (5000 resamples), which provides a more accurate confidence interval (CI) for mediation analysis than the Sobel test or stepwise regression *p*-values. This approach effectively avoids the high Type I error rate associated with the causal steps method, and is particularly suitable for testing complex models. Table 2 reports the regression results of the main and mediating effect tests.

To test the effect of empowering leadership on job crafting, we incorporated the control variables of gender, age, educational background, working years, position level, and the independent variable of empowering leadership into regression model M1 of job crafting. The regression results revealed that empowering leadership had a significantly positive effect on job crafting (Î^2^ = 0.56, *p* < 0.001). Thus, Hypothesis 1 was verified. The effect of role breadth self-efficacy on job crafting is shown in Model M3, which confirming that role breadth self-efficacy had a significantly positive effect on job crafting (β = 0.38, *p* < 0.001). Thus, Hypothesis 3 was verified.

To test the mediating effect of empowering leadership on job crafting through role breadth self-efficacy, we constructed Model M2 based on the main effect of Model M1. The results showed that empowering leadership had a significantly positive effect on role breadth self-efficacy (β = 0.42, *p* < 0.001). Thus, Hypothesis 2 was verified. In addition, regression Model M4, which contains control variables, empowering leadership, and role breadth self-efficacy affecting job crafting, shows that role breadth self-efficacy has a significantly positive effect on job crafting (β = 0.16, *p* < 0.001), and the effect of empowering leadership on job crafting decreases from 0.56 Model M1 0.49 Model M4. These regression results provide initial support for the mediation model, which was further rigorously tested using bootstrapping. To rigorously verify the mediation effect, we examined the indirect effect using bias-corrected bootstrapping with 5000 resamples using PROCESS Model 4. The results indicate that the indirect effect of empowering leadership on job crafting via role breadth self-efficacy was significant, with a 95% confidence interval of [0.386, 0.609], which did not include zero. Therefore, the mediating role of role breadth self-efficacy was confirmed, thus supporting Hypothesis 4.

To test the moderating effect (H5) and moderated mediation effect (H6), we utilized Model 7 of the PROCESS macro. As shown in Table 3 (Model M5), the interaction term empowering leadership × learning-goal orientation has a significantly positive effect on role breadth self-efficacy (β = 0.176, *p* < 0.001). Hypothesis 5 was verified, indicating that learning-goal orientation can significantly enhance the positive effect of empowering leadership on role-breadth self-efficacy.

**Figure 3:**
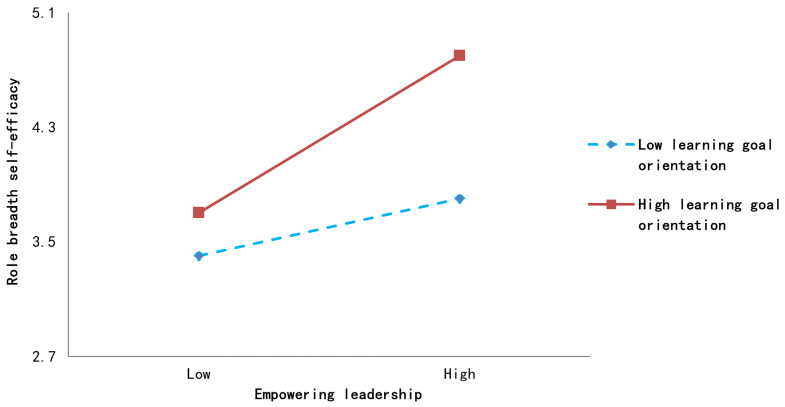
Moderating effects of role breadth self-efficacy.

Additionally, we further categorized learning goal orientation into high-scoring (mean + 1 standard deviation) and low-scoring groups (mean − 1 standard deviation) to construct the moderation effect diagram shown in Figure 3 (Cohen, 2003). Empowering leadership exerts a stronger influence on role breadth self-efficacy when employees exhibit high levels of learning-goal orientation. Conversely, when employees demonstrate low levels of learning-goal orientation, empowering leadership exerts a weaker influence on role breadth self-efficacy.

To test the mediating effect at different levels of learning-goal orientation, we set bootstrap resampling 5000 times. The results show that the 95% confidence interval for the moderated mediating effect was [0.005–0.06], as shown in Table 4. When learning goal orientation was one standard deviation greater than the mean, the mediating effect of role breadth self-efficacy between empowering leadership and job crafting behavior was significant; the conditional mediating effect was 0.0817, and the 95% confidence interval was [0.3705, 0.6756]. When the level of learning-goal orientation was one standard deviation below the mean, the mediating effect was still significant, with a 95% confidence interval of [0.0014, 0.2428]; however, the conditional mediating effect was significantly reduced. That is, as the level of learning-goal orientation increased, the effect of empowering leadership on job crafting through role breadth self-efficacy became stronger, thus verifying Hypothesis 6.

## 5. Discussion

The primary objective of this study was to explore the mechanism linking empowering leadership to knowledge workers’ job crafting. The empirical results support our moderated mediation model. Specifically, we found that empowering leadership significantly promotes job crafting. This finding aligns with previous studies in Western contexts (e.g., Kim & Beehr, 2018), confirming that autonomy-supportive behaviors are universal drivers of proactive work behaviors. Furthermore, role breadth self-efficacy serves as a crucial mediator. This result supports Parker’s (1998) proposition that environmental enablers (leadership) must first enhance individuals’ perceived capability (self-efficacy) to foster broader role orientations. Finally, learning goal orientation positively moderates the first stage of this mediation process, amplifying the cognitive activation effect of empowerment. This echoes VandeWalle’s (1997) goal orientation theory, suggesting that individuals with a growth mindset are more receptive to the resources provided by empowerment.

### 5.1. Limitations

Despite its contributions, this study had several limitations that suggest directions for future research. First, its cross-sectional design limits our ability to make definitive causal inferences. Although our theoretical model is grounded in causal logic, future research should employ longitudinal or experimental designs to verify the causality between empowerment and crafting rigorously. Second, all variables were self-reported by employees, which may introduce common method bias. While statistical tests (Harman’s single-factor test) indicated no serious bias, future studies could benefit from multi-source data collection (e.g., supervisor ratings) (Podsakoff et al., 2003). Third, the sample was drawn exclusively from Chinese knowledge workers. Given the unique high-power distance culture in China, the generalizability of these findings to Western low-power-distance cultures requires further empirical validation.

### 5.2. Theoretical Implications

This study makes three major theoretical contributions. First, it moves beyond the traditional examination of leadership effectiveness to reveal the cognitive mechanisms underlying knowledge workers’ proactive behavior (Bruning & Campion, 2018). Unlike previous research, which predominantly viewed job crafting as a direct outcome of personality traits (Bateman & Crant, 1993), our findings clarify that environmental factors (empowering leadership) must be translated into internal psychological resources (role breadth self-efficacy) to drive behavioral change (Zhang & Bartol, 2010). This confirms the triadic reciprocal determinism of Social Cognitive Theory (Bandura, 2001), identifying role breadth self-efficacy as a critical “cognitive bridge” that transforms structural empowerment into psychological motivation. Second, this study integrates the “contingency perspective” by identifying learning-goal orientation as a key boundary condition. We found that the effectiveness of empowering leadership is not universal but depends on employees’ intrinsic motivational orientation (Lee et al., 2018). Highly learning-oriented individuals are more capable of interpreting empowerment as a developmental opportunity rather than a burden, thereby extracting greater cognitive benefits from leadership support. Third, considering the Chinese cultural context, these findings offer unique cultural insights. China is traditionally characterized as a high-power distance society (Hofstede, 2001), where employees typically depend on hierarchy. Unlike in low-power distance cultures where participation is expected, in such a context, empowering leadership acts as a positive “deviant” signal. This explicit signal of trust creates a stronger “contrast effect” than in Western cultures (Zhang & Bartol, 2010), effectively breaking traditional hierarchy dependency and efficiently activating employees’ self-efficacy.

### 5.3. Practical Implications

These findings imply that organizations should move beyond standardized management. First, managers should implement structural empowerment by granting knowledge workers genuine autonomy in project management and decision making. Second, fostering a psychologically safe environment is crucial to protect the “cognitive bridge” of self-efficacy (Edmondson, 1999); managers must support experimentation and reduce the fear of failure. Third, differentiated management strategies are necessary. For employees with a high learning goal orientation, leaders should provide challenging tasks to maximize their growth potential; for those with a low learning orientation, leaders should offer more guidance to reduce role ambiguity and resistance to empowerment.

## 6. Conclusions

This study clarifies how and when empowering leadership drives job crafting among knowledge workers. By identifying role breadth self-efficacy as the cognitive engine and learning goal orientation as the motivational catalyst, our model highlights that effective leadership is not just about giving power but about activating the internal psychological capacity of employees. These insights provide a robust theoretical basis for organizations that aim to foster an adaptive and proactive workforce in the digital economy.

## Figures and Tables

**Table 1:** Descriptive statistics and correlation analysis (N = 338).

Variable	Mean	SD	1	2	3
1. Empowering leadership	3.60	1.05			
2. Job crafting	3.48	1.16	0.51 ***		
3. Role breadth self-efficacy	3.68	1.05	0.43 ***	0.34 **	
4. Learning goal orientation	3.78	0.98	0.45 ***	0.46 ***	0.47 ***

Note: *** *p* < 0.001; ** *p* < 0.01.

**Table 2:** Main effect and mediating effect tests. (Testing H1, H2, H3, and H4).

	M1	M2	M3	M4
Gender	−0.20	−0.13	−0.21	−0.18
Age	−0.03	−0.03	−0.02	−0.02
Educational background	−0.05	0.02	−0.05	−0.05
Working years	0.11	−0.01	0.12	0.11
Position level	0.07	−0.04	0.10	0.07
Empowering leadership	0.56 ***	0.42 ***		0.49 ***
Role breadth self-efficacy			0.38 ***	0.16 **
R^2^	0.28	0.19	0.14	0.30
ΔR^2^	0.27	0.19	0.14	0.30
F	21.54	13.00	8.85	20.05

Note: *** *p* < 0.001; ** *p* < 0.01.

**Table 3:** Test of moderating effects. (Testing H5).

Variable	M5
Gender	−0.107
Age	−0.027
Educational background	0.008
Working years	−0.017
Position level	−0.039
Empowering leadership	0.322 ***
Learning goal orientation	0.303 ***
Empowering leadership × Learning goal orientation	0.176 ***
R^2^	0.323
ΔR^2^	0.323
F	19.634

Note: *** *p* < 0.001.

**Table 4:** Conditional indirect effects at specific levels of the moderator (Testing H6).

The Level of Learning Goal Orientation	Conditional Mediating Effect	BootSE	Boot LLCI	Boot ULCI
eff1(M − 1SD)	0.1221	0.0614	0.0014	0.2428
eff2(M)	0.3226	0.0518	0.2207	0.4244
eff3(M + 1SD)	0.5230	0.0776	0.3705	0.6756

## Data Availability

The data presented in this study are available upon request from the corresponding authors. The data were not publicly available because of privacy concerns.
